# Supplementary material for: An Arabic Sedentary Behaviors Questionnaire (ASBQ): Development, Content Validation, and Pre-Testing Findings
Source: Behav Sci (Basel). 2022 Jun 8;12(6):183. doi: 10.3390/bs12060183 (PMC9220100; doi:10.3390/bs12060183)
Supplement: Supplementary file 1 [file behavsci-12-00183-s001.zip › Table S1- Searched studies .pdf]

Table S1. Detailed description of the characteristics of the searched studies.

ID	Questionnaire Name	Questionnaire abbreviation	Authors, year	# items	population				Sample size	Note
					Age	Gender	Condition	Country		
**1**	Adult Sedentary Behavior Questionnaire	ASBQ	Chu, et al., 2018 (ref # 36)	7	21–65 years	Both	English-literate healthy adult	Singaporean adults of Chinese, Malay, & Indians ethnicity	84	Against single item GPAQ, accelerometer ADAPTED from SBQ
**2**	Adolescent Sedentary Activity Questionnaire	ASAQ	Hardy, et al., 2007 (ref #44)		11 -15 years	Both	school classes of adolescents	Australia	250	none
**3**	Time spent sitting on weekdays and weekend days	Daily SB	Marshall, et al., 2010 (ref # 31)	5: Traveling, at work, TV viewing, computer use for home & for leisure	51-59 years women 45-63 years men	Both	healthy adults	Australia	157 women96 men	gender-specific test-retest reliability and validity
**4**	Occupational sitting, standing, & physical activity time	OSPAQ	Chau, et al., 2012 (ref # 29)	6	> 18 years	Both	English-literate healthy adults	Australia	99	Against accelerometer
**5**	Physical Activity and Sedentary Behaviour Questionnaire	PASB-Q	Fowles, et al., 2017 (ref # 41)	7	> 18 years	Both	healthy	Canada	35	Against accelerometer
**6**	Past-day Adults' Sedentary Time	PAST	Clark, et al., 2013 (ref # 30)	7	18 – 75 years	female	diagnosed with breast cancer (stages I–	Australia	90	Against inclinometer and accelerometer

							III), BMI 25 - 40			
**7**	Sedentary Behavior Questionnaire	SBQ	Rosenberg, et al., 2010 (ref # 32)	9	> 18	Both	Healthy or overweight & obese	USA	49 + 842	Against IPAQ and accelerometer
**8**	SIT-Q	SIT-Q	Lynch, et al., 2014 (ref # 46)	7 sections	30-60 years	Both	Healthy, BMI > 35	Canada	82	Against 7-Day activity diary
**9 & 10**	last 7-d sedentary behavior questionnaire	SIT-Q-7d	Felez-Nobrega M, et al, 2019 (ref # 40) & Wijndaele, et al., 2014 (ref # 33)	5 sections	20-60 & 40-65	Both	Healthy, at risk for diabetes	Belgium & UK	51 + 402	Against ActivPAL, Actiheart Adapted from SIT-Q
**11**	Sedentary Time and Activity Reporting Questionnaire	STAR-Q	Csizmadi, et al., 2014 (ref # 37)	15 sections	30-60 years	Both	Healthy, BMI > 35	Canada	91	Against 7-Day activity diary
**12**	Sedentary, Transportation and Activity Questionnaire	STAQ	Mensah, et al., 2016 (ref # 48)	5 sections	20- 65 years	Both	healthy	France	96	Against accelerometer
**13**	Modified Worker's Living Activity-time Questionnaire	mWALQ	Matsuo, et al., 2020 (ref # 47)	15	30-60 years	Both	Healthy working adults	Japan	198	Against VO2max measurement
**14**	Youth Leisure-time Sedentary Behavior Questionnaire	YLSBQ	Cabanas-Sánchez, et al.,	15	11-15 years	Both	Healthy, school classes of	Spain	208, 1638	Against accelerometer

			2018 (ref # 35)				adolescents			
**15**	Self-Administered Physical Activity Checklist	3-Day SAPAC	Affuso, et al., 2011 (ref # 34)		11-15 years	Both	Healthy, school classes of adolescents	USA	224	Against accelerometer
**16**	Physical Activity and Sedentary Behavior Assessment Questionnaire	PASBAQ	Scholes, et al., 2013 (ref # 49)	4 domains	≥16 years	Both	living in English households	UK	2175	Against accelerometer
**17**	Multimedia Activity Recall for Children and Adults	MARCA	Gomersall, et al., 2015 (ref # 42)	7 categories	-	Both	healthy	Australia	58	Against ActivPAL
**18**	Transtheoretical Model questionnaire for sedentary behaviors	TTM	Han, et al., 2015 (ref # 43)	10 processes of change (40 items)	18 – 24 years	Both	College students	USA	225	Against accelerometer
**19**	Multicontext Sitting Time Questionnaire	MSTQ	Whitfield, et al., 2013 (ref # 50)	7 domains for working and non-working time	Mean (sd) = 34.5 (7.7)	Both	Recreational runners	USA	25	ActiGraph GT1M accelerometer
**20**	South American Youth Cardiovascular and Environmental sedentary behavior questionnaire	SAYCARE	De Moraes, et al., 2020 (ref # 38)	4 domains	Childre: 3-10 Adolescents: 11-18	Both	Healthy school children	SAmerica (Argentina, Peru, Colombia, Uruguay, Chile & Brazil)	400 for both validity and reliability	Validity was Against accelerometer
**21**	TAxonomy of Self-report SB Tools	TASST	Dontje, et al.,	4 domains	65 + years	Both	Elderly	United Kingdom	18	activPAL 3c

			2018 (ref # 39)							
**22**	Child screen media use questionnaire	SCREENS-Q	Klakk, et al., 2020 (ref # 45)	6 domains	6 –10-year old children	Both	Children	Denmark	37 parents	Content validity and reliability
