# Supplementary material for: An Arabic Sedentary Behaviors Questionnaire (ASBQ): Development, Content Validation, and Pre-Testing Findings
Source: Behav Sci (Basel). 2022 Jun 8;12(6):183. doi: 10.3390/bs12060183 (PMC9220100; doi:10.3390/bs12060183)
Supplement: Supplementary file 1 [file behavsci-12-00183-s001.zip › Table S2- Arabic SBQ .pdf]

Table S2: Arabic sedentary behaviors questionnaire (ASBQ)

استبانة قياس السلوك الخامل (ASBQ)

يهدف هذا الاستبيان إلى قياس مقدار وقت القعود (أو الجلوس) خلال كامل أيام الأسبوع، لذا نرجو منكم التكرم بالإجابة على الأسئلة بكل دقة وصدق، علما بأن السلوك الخامل يعني أي سلوك يتضمن القعود (أو الجلوس) أو الاستلقاء خلال اليوم، ولا يدخل ضمن ذلك فترات النوم. حاول أن تجيب على مقدار سلوك القعود الأسبوعي المعتاد لك، وليس خلال هذا الأسبوع فقط والذي قد لا يمثل نمط سلوك القعود المعتاد لك، كما في حالة وجود مرض أو ظرف طارئ كاختبار أو ملازمة مريض مثلاً، مما يجعل نمط سلوكك يتغير في هذه الحال.

*** من يعمل أو يذهب للمدرسة عليه الإجابة على الجزئين أولاً وثانياً، ومن لا يعمل أو متقاعد فعليه الإجابة على الجزء ثانياً.**

أولاً: كم المدة التي تقضيها في ممارسة السلوكيات التالية أثناء ساعات المدرسة أو العمل؟ (من لا يعمل أو متقاعد: فضلاً أنتقل مباشرة إلى ثانياً). أجب حسب معدل الوقت الذي تقضيه أثناء أيام الأسبوع المعتادة (غير أيام نهاية الأسبوع).

1 . كم يومًا في الأسبوع عادة تذهب فيها إلى المدرسة أو العمل؟ من لا يعمل أو متقاعد: أنتقل مباشرة إلى ثانياً	[ ] أيام
2 . في المعتاد، كم المدة في اليوم التي تقضيها في القعود (أو الجلوس) سواء في المدرسة أو العمل؟	ساعة [ ] دقيقة [ ]

ثانياً: كم هي المدة التي تقضيها في ممارسة السلوكيات التالية؟ أجب حسب معدل الوقت الذي عادة تقضيه في ممارسة هذه الأنشطة أثناء أيام الأسبوع ونهاية الأسبوع. أحسب كل فترة جلوس مرة واحدة فقط حسب النشاط الرئيسي لها. على سبيل المثال، إذا جلست على الأريكة (الكنبة أو الكرسي) وقرأت كتابًا لمدة ساعة واحدة بينما كنت تستمع للقرآن، فاحسب هذه الساعة كساعة واحدة قضيتها في القراءة إن كانت القراءة هي النشاط الرئيسي لهذه الجلسة، أي لا تحسب هذه الساعة ضمن النشاط الآخر وهو الاستماع للقرآن.

السلوك أو النشاط	خلال أيام الأسبوع	خلال يومي نهاية الأسبوع
3. مشاهدة الأفلام، أو التلفاز، أو مقاطع الفيديو (بغض النظر عما إذا كان مصدرها التلفاز أو جهاز الكمبيوتر أو الهاتف النقال)	ساعة [ ] دقيقة [ ]	ساعة [ ] دقيقة [ ]
4. تصفح الإنترنت أو استخدام مواقع التواصل الاجتماعي بغرض التسلية، ومن أي جهاز كان	ساعة [ ] دقيقة [ ]	ساعة [ ] دقيقة [ ]
5. ممارسة الألعاب الإلكترونية من وضع القعود (أو الجلوس أو الاستلقاء) ومن أي جهاز كان	ساعة [ ] دقيقة [ ]	ساعة [ ] دقيقة [ ]
6. القيام بالواجبات المنزلية الدراسية للطالب (أو الواجبات الوظيفية المنزلية للموظف) بطريقة تقليدية أو الكترونية	ساعة [ ] دقيقة [ ]	ساعة [ ] دقيقة [ ]
7. القراءة بغرض التسلية	ساعة [ ] دقيقة [ ]	ساعة [ ] دقيقة [ ]
8. التحدث مع أفراد الأسرة أو الأصدقاء وجهًا لوجه أو عبر الإنترنت	ساعة [ ] دقيقة [ ]	ساعة [ ] دقيقة [ ]
9. الاستماع للقرآن أو الراديو أو الموسيقى (دون القيام بأي نشاط آخر في الوقت نفسه)	ساعة [ ] دقيقة [ ]	ساعة [ ] دقيقة [ ]
10. أخذ قسطاً من الراحة (كالاستلقاء بغرض الاسترخاء) وليس النوم	ساعة [ ] دقيقة [ ]	ساعة [ ] دقيقة [ ]
11. ممارسة الهوايات التي تتطلب التفكير أو التعليل أو الاستنتاج (مثل حل الأحجيات والألغاز، لعب الورق، حل الكلمات المتقاطعة، وغيرها)	ساعة [ ] دقيقة [ ]	ساعة [ ] دقيقة [ ]
12. ممارسة الهوايات اليدوية أثناء الجلوس (كالرسم، الحياكة، الخياطة، أعمال فنية أخرى، وغيرها)	ساعة [ ] دقيقة [ ]	ساعة [ ] دقيقة [ ]
13. استخدام وسائل المواصلات التي تتطلب الجلوس (سواء في السيارة، أو الحافلة، أو القطار، أو قطار الأنفاق، أو الدراجة النارية) خلال اليوم	ساعة [ ] دقيقة [ ]	ساعة [ ] دقيقة [ ]

14. القيام بالأعمال المنزلية بينما تكون جالسًا (كي الملابس وطيّها، أو تقطيع الخضار والفواكه، أو تصليح الأدوات، وغيرها)	ساعة [ ] دقيقة [ ]	ساعة [ ] دقيقة [ ]
15. ممارسة أنشطة أخرى، غير ما تم ذكره أعلاه، بينما تكون جالسًا أو مستلقياً. فضلاً اذكرها وسجّل مجموع الوقت:	ساعة [ ] دقيقة [ ]	ساعة [ ] دقيقة [ ]
16. كم هي المدة التي عادة ما تقوم خلالها بقطع مدة جلوسك لبضع دقائق في اليوم، من خلال الوقوف أو المشي في مكان ما، أو الحركة مشيًا للحصول على الشاي أو القهوة؟		
□ كل ٣٠ دقيقة أو أقل □ كل ساعة □ كل ساعتين □ كل ٣ ساعات □ كل ٤ ساعات أو أكثر		

شكراً لحسن تجاوبكم
