# Supplementary material for: An Arabic Sedentary Behaviors Questionnaire (ASBQ): Development, Content Validation, and Pre-Testing Findings
Source: Behav Sci (Basel). 2022 Jun 8;12(6):183. doi: 10.3390/bs12060183 (PMC9220100; doi:10.3390/bs12060183)
Supplement: Supplementary file 1 [file behavsci-12-00183-s001.zip › Table S3- Translated Arabic Sedentary Behavior Questionnaire (ASBQ) .pdf]

Table S3. English translation of Arabic Sedentary Behavior Questionnaire (ASBQ)

Sedentary behaviors are any waking behavior that involves sitting, lying or reclining position. When asked about sedentary behaviors, you must think about behaviors that meet these characteristics. **For those who are working or students, kindly answer both sections 1 and 2. For those who are unemployed or retired answer section 2 only**.

Section 1:

- **During SCHOOL/WORK HOURS**, How many hours per day do you spend sitting **during school/work hours**? Respond thinking about the **average** time you spend during a typical (usual) week.

1. How many **days per week** do you typically **go to** work or school?	[ ] days
2. How many **hours per day do you spend** sitting while at work or school?	[ ] hrs. [ ] min

Section 2:

- **How long have you been engaged in the following behaviors during weekdays or weekends?**

Respond to the following questions thinking about AVERAGE time you spend on each activity during a typical (usual) weekdays or weekends. **Each period of sitting down should only be entered once**. For example, if you spent one hour sitting on the sofa reading a book while you were listening to music, count this time as one hour reading if this was your main activity. Do not count this as one hour reading a book and another hour listening to music.

**How many** hours and min per day you spend **sitting, lying or reclining while:**	**During weekdays**	**During weekends**
3. Watching movies, TV, videos (regardless of the source-TV, computer, phone)	[ ] hrs. [ ] min	[ ] hrs. [ ] min
4. Playing computer/video games	[ ] hrs. [ ] min	[ ] hrs. [ ] min
5. Internet surfing or using social media for fun	[ ] hrs. [ ] min	[ ] hrs. [ ] min
6. Doing homework/studying	[ ] hrs. [ ] min	[ ] hrs. [ ] min
7. Reading for fun	[ ] hrs. [ ] min	[ ] hrs. [ ] min
8. Sitting and talking with family or friends in person or via internet	[ ] hrs. [ ] min	[ ] hrs. [ ] min
9. Listening to Quran, Radio, or music (without doing anything else)	[ ] hrs. [ ] min	[ ] hrs. [ ] min
10. Resting (lying down, but not taking a nap, etc.)	[ ] hrs. [ ] min	[ ] hrs. [ ] min
11. Doing hobbies that require thinking/reasoning (doing puzzles, playing cards, doing crossword puzzles, etc.)	[ ] hrs. [ ] min	[ ] hrs. [ ] min
12. Doing crafts or art work <u>while sitting</u> (like drawing, knitting, sewing, etc.)	[ ] hrs. [ ] min	[ ] hrs. [ ] min
13. Using transportation while sitting (in car, bus, train, subway or motorbike)	[ ] hrs. [ ] min	[ ] hrs. [ ] min
14. Doing household tasks <u>while seated</u> (ironing, slicing foods, repairing things)	[ ] hrs. [ ] min	[ ] hrs. [ ] min
15. Engaged in other activities than the above <u>while seated</u>, name them: ……	[ ] hrs. [ ] min	[ ] hrs. [ ] min

16. Typically, how often do you **interrupt your sitting** time during a typical day in a week?				
□ every 30 min or less	□ every 1 hour	□ every 2 hours	□ every 3 hours	□ every 4 hours or more

Thank you for participating in this survey.
